# Supplementary material for: Accurate Encoding and Decoding by Single Cells: Amplitude Versus Frequency Modulation
Source: PLoS Comput Biol. 2015 Jun 1;11(6):e1004222. doi: 10.1371/journal.pcbi.1004222 (PMC4452646; doi:10.1371/journal.pcbi.1004222)
Supplement: S1 Text — (PDF) [file pcbi.1004222.s001.pdf]

S1 Text for
Accurate encoding and decoding by single cells: amplitude versus frequency modulation

Gabriele Micali,[1,2,3] Gerardo Aquino,[1,2] David M. Richards,[1,2] and Robert G. Endres[1,2,*]
[1]*Department of Life Sciences, Imperial College, London, UK*
[2]*Centre for Integrative System Biology and Bioinformatics, Imperial College, London, UK*
[3]*Dipartimento di Fisica, Università degli Studi di Milano, Milano, Italy*

CONCENTRATION SENSING BY CM RECEPTOR

In this section we calculate the analytic solution for the master equation (Eq. 1a,b in the main text) for continuous modulation (CM). For clarity, we repeat here the master equations for the *on* and *off* states:

$$\frac{dp_{\text{on}}(n,t)}{dt} = \gamma(n+1)p_{\text{on}}(n+1,t) + \alpha p_{\text{on}}(n-1,t) + k_+c\, p_{\text{off}}(n,t) - (\gamma n + \alpha + k_-)p_{\text{on}}(n,t),$$
$$\frac{dp_{\text{off}}(n,t)}{dt} = \gamma(n+1)p_{\text{off}}(n+1,t) + k_- p_{\text{on}}(n,t) - (\gamma n + k_+c)p_{\text{off}}(n,t),$$

where $p_{\text{on/off}}(n,t)$ is the probability that the receptor/ion channel is in the *on*/*off* state with n output proteins at time t, α and γ are the production and degradation rates respectively, and k_+c and k_- are the binding and unbinding rates respectively. Note that the concentration of the input species (c) is now constant. Eqs. (1a,b) can be rewritten as Eq. (15) in the main text

$$\frac{dp_s(n,t)}{dt} = \gamma(n+1)p_s(n+1,t) + \alpha_s p_s(n-1,t) + k_{\bar{s}}p_{\bar{s}}(n,t) - (\gamma n + \alpha_s + k_s)p_s(n,t),$$

where $\bar{s}$ is the *on*/*off* state when s is the *off*/*on* state, and $\alpha_{\text{on}} = \alpha$, $\alpha_{\text{off}} = 0$, $k_{\text{on}} = k_-$ and $k_{\text{off}} = k_+c$. To find the solution for the first two moments of the distribution $p(n,t)$, we now follow Mehta and Schwab [62]. At steady state Eq. (15) becomes

$$K_{\bar{s}}p_{\bar{s}}(n) = -(n+1)p_s(n+1) - A_s p_s(n-1) + (n + A_s + K_s)p_s(n), \tag{S2}$$

where $K_s = k_s/\gamma$ and $A_s = \alpha_s/\gamma$. Using the generating function in Eq. (16)

$$G_s(z) = \sum_{n=0}^{\infty} p_s(n) z^n,$$

Eq. (S2) becomes

$$\left[(z-1)\partial_z - A_s(z-1) + K_s\right] G_s(z) = K_{\bar{s}}, \tag{S3}$$

which implies

$$(\partial_z - A_{\text{on}})\, G_{\text{on}} = \frac{K_{\text{off}}\; G_{\mathit{off}} - K_{\text{on}}\; G_{\mathit{on}}}{z-1}, \tag{S4}$$
$$(\partial_z - A_{\text{off}})\, G_{\text{off}} = \frac{K_{\text{on}}\; G_{\mathit{on}} - K_{\text{off}}\; G_{\mathit{off}}}{z-1}, \tag{S5}$$

which, when combined, gives

$$(\partial_z - A_{\text{on}})\, G_{\text{on}} = -\,(\partial_z - A_{\text{off}})\, G_{\text{off}}. \tag{S6}$$

* r.endres@imperial.ac.uk

To proceed further, it is useful to define the quantity $H_s(z)$ related to the generating function $G_s(z)$ by

$$G_s(z) = e^{A_s z} H_s(z). \tag{S7}$$

Using

$$\Big(\partial_z - A_s\Big) G_s(z) = e^{A_s z}\partial_z H_s(z), \tag{S8}$$

Eq. (S6) becomes

$$e^{A_{\rm on} z}\partial_z H_{\rm on}(z) = -e^{A_{\rm off} z}\partial_z H_{\rm off}(z), \tag{S9}$$

which links the expressions for $H_{\rm on}$ and $H_{\rm off}$. At this point the initial equation for the steady state (Eq. (S3)) becomes

$$(z-1)e^{A_s z}\partial_z H_s(z) + K_s e^{A_s z}\partial_z H_s(z) = K_{\bar{s}} e^{A_{\bar{s}} z}\partial_z H_{\bar{s}}(z). \tag{S10}$$

Multiplying by $e^{A_{\bar{s}}}$, taking the derivative with respect to z, substituting Eq. (S9), and defining $\Delta A_s = A_{\bar{s}} - A_s$, gives

$$\partial_z H_s(z) - \Delta A_s (z-1)\partial_z H_s(z) + (z-1)\partial_z^2 H_s(z) - K_s \Delta A_s H_s(z) + K_s \partial_z H_s(z) = -K_{\bar{s}} H_s(z)$$

and hence

$$(z-1)\partial_z^2 H_s(z) + \Big(1 - \Delta A_s(z-1) + K_s + K_{\bar{s}}\Big)\partial_z H_s(z) - K_s \Delta A_s H_s(z) = 0.$$

Finally, changing variables to $u = \Delta A_s (z-1)$ provides

$$u\partial_z^2 H_s(u) + (1 + K_s + K_{\bar{s}} - u)\,\partial_z H_s(u) - K_s H_s(u) = 0. \tag{S11}$$

This is the confluent hypergeometric equation, for which the solution in terms of confluent hypergeometric functions of the first kind is given by

$$H_s(u) = c_s \; {}_1F_1(K_s, 1 + K_s + K_{\bar{s}}; u)\,, \tag{S12}$$

with c_s a constant of integration. Thus, through Eq. (S7),

$$G_s(z) = c_s e^{A_s z} \; {}_1F_1(K_s, 1 + K_s + K_{\bar{s}}; \Delta A_s (z-1))\,. \tag{S13}$$

To determine the constants, notice that ${}_1F_1(a, b, 0) = 1$ leading to

$$G_s(1) = c_s e^{A_s} = \langle p_s \rangle = \frac{K_{\bar{s}}}{K_{\bar{s}} + K_s},$$

where $\langle p_s \rangle$ is the average probability of being in state s. Rearranging terms, we obtain

$$c_s = \frac{K_{\bar{s}}}{K_{\bar{s}} + K_s} e^{-A_s}. \tag{S14}$$

Finally, the probability distribution at steady state is given by [62]

$$G_s(z) = \frac{K_{\bar{s}} e^{A_s (z-1)}}{K_{\bar{s}} + K_s} \; {}_1F_1(K_s, 1 + K_s + K_{\bar{s}}; \Delta K_2^s (z-1))\,. \tag{S15}$$

Having an analytic expression for the steady-state probability distribution (Eq. S15), we can now calculate the first, second and third moments, which are related to the mean, variance and skewness, respectively. The mean production of the output protein is given by the mean production in the *on* state multiplied by the probability to be in the *on* state, averaged over the whole time period. For such a two-state system $\langle p_{\rm on} \rangle = \frac{K_{\rm off}}{K_{\rm off} + K_{\rm on}}$ and $\langle p_{\rm off} \rangle = 1 - \langle p_{\rm on} \rangle$. Therefore, the mean number of proteins is given by

$$\langle n \rangle = (A_{\rm on} - A_{\rm off})\,\langle p_{\rm on} \rangle + A_{\rm off} = \frac{\alpha}{\gamma}\frac{k_+ c/k_-}{1 + k_+ c/k_-}. \tag{S16}$$

To calculate the variance, we use the following property of the generating function:

$$(\delta n)^2 = \sum_s \left(\partial_z z \partial_z G_s(z)\right)\big|_{z=1} - \langle n \rangle^2\,. \tag{S17}$$

Proof.

$$
\begin{aligned}
(\delta n)^2 &= \sum_s \left[\sum_n n^2 p_s(n)\right] - \langle n\rangle \\
&= \sum_s \left[\sum_n n^2 p_s(n)\right]_{z=1} - \langle n\rangle \\
&= \sum_s \left[\sum_n z\partial_z \left(n p_s(n) z^n\right) n^2 p_s(n)\right]_{z=1} - \langle n\rangle \\
&= \sum_s \left[z\partial_z z\partial_z G_s(z)\right]_{z=1} - \langle n\rangle \\
&= \sum_s \left[\partial_z z\partial_z G_s(z)\right]_{z=1} - \langle n\rangle .
\end{aligned}
$$

□

Using common properties of hypergeometric functions, the analytical solution for the variance is [62]

$$
\left\langle (\delta n)^2 \right\rangle = \langle n\rangle + \langle p_{\text{on}}\rangle \langle p_{\text{off}}\rangle \frac{(\Delta A_s)^2}{1+K_s+K_{\bar{s}}} = \langle n\rangle + \frac{\alpha^2}{\gamma\left(\gamma+k_-+k_+c\right)} \frac{k_+c/k_-}{\left(1+k_+c/k_-\right)^2}. \tag{S18}
$$

For details, see the full calculation in the SI of [62].

Third moment

In order to understand more about the symmetry of the probability distribution, we calculate the third moment at steady state. As in Eq. (S17) the third moment can be found via generating functions as

$$
\langle n^3\rangle = \sum_s \left[\partial_z z\partial_z z\partial_z G_s(z)\right]\Big|_{z=1} = \sum_s \left[3z\partial_z^2 G_s(z) + z^2\partial_z^3 G_s(z) + \partial_z G_s(z)\right]\Big|_{z=1}, \tag{S19}
$$

where [62]

$$
\sum_s \partial_z G_s(z)\Big|_{z=1} = \langle n\rangle , \tag{S20}
$$

$$
\begin{aligned}
\sum_s \left(z\partial_z^2 G_s(z)\right)\Big|_{z=1} &= \langle p_{\text{on}}\rangle (A_{\text{on}})^2 + \langle p_{\text{off}}\rangle (A_{\text{off}})^2 - \langle p_{\text{on}}\rangle \langle p_{\text{off}}\rangle \frac{\Delta A_s (K_{\text{on}}+K_{\text{off}})}{1+K_s+K_{\bar{s}}} \\
&= \frac{\alpha^2 (k_+c/k_-)}{\gamma^2(1+k_+c/k_-)} + \frac{\alpha(k_+c)}{\gamma(1+k_+c/k_-)(\gamma+k_-+k_+c)}.
\end{aligned} \tag{S21}
$$

Thus, only $\sum_s z^2\partial_z^3 G_s(z)\big|_{z=1}$ needs to be calculated. The result is

$$
\begin{aligned}
\sum_s \left(z^2\partial_z^3 G_s(z)\right)\Big|_{z=1} &= (A_{\text{on}})^3 \langle p_{\text{on}}\rangle + (A_{\text{off}})^3 \langle p_{\text{off}}\rangle - \frac{\langle p_{\text{on}}\rangle \langle p_{\text{off}}\rangle (\Delta A_{\text{on}})^2 \left(K_{\text{on}}+K_{\text{off}}\right)}{1+K_{\text{on}}+K_{\text{off}}} \\
&\quad \times \left[\frac{\left(3+K_{\text{on}}+2K_{\text{off}}\right) A_{\text{on}}}{2+K_{\text{on}}+K_{\text{off}}} + \frac{\left(3+2K_{\text{on}}+K_{\text{off}}\right) A_{\text{off}}}{2+K_{\text{on}}+K_{\text{off}}}\right] \\
&= \frac{\alpha^3(k_+c/k_-)}{\gamma^3(1+k_+c/k_-)} - \frac{\alpha^3(k_+c/k_-)\left(3\gamma+k_-+2k_+c\right)}{\gamma^3(1+k_+c/k_-)\left(\gamma+k_-+k_+c\right)\left(2\gamma+k_-+k_+c\right)}.
\end{aligned} \tag{S22}
$$

By combining Eqs. (S57)-(S59) as indicated in Eq. (S56), we obtain the analytic expression for the skewness of our system.

Proof. From the definition of confluent hypergeometric functions of the first kind

$$ {}_1F_1(a,b;z) = \sum_{n=0}^{\infty} \frac{a_n}{b_n n!} z^n, $$

with

$$ a_n = a(a+1)(a+2)...(a+n-1), $$
$$ b_n = b(b+1)(b+2)...(b+n-1), $$

and the fact that

$$ z\partial_z\ {}_1F_1(a,b;z) = z\frac{a}{b}\ {}_1F_1(a+1,b+1;z), \tag{S23} $$

we obtain

$$ \partial_z G_s(z) = \frac{K_{\bar{s}}}{K_{\bar{s}}+K_s}\Big[A_s e^{A_s(z-1)}\ {}_1F_1\left(\cdot,\cdot,\Delta A_s(z-1)\right) + \frac{\Delta A_s K_s}{1+K_s+K_{\bar{s}}} e^{A_s(z-1)}\ {}_1F_1\left(+,+,\Delta A_s(z-1)\right)\Big], \tag{S24} $$

where $\Delta A_s = A_{\bar{s}} - A_s$ and ${}_1F_1(+,+,z) =\ {}_1F_1(a+1,b+1,z)$. We now need to calculate $\sum_s z^2\partial_z^3 G_s(z)\big|_{z=1}$. Using Eq. (S21) we find that

$$ \begin{aligned} \left(z^2\partial_z^3 G_s(z)\right)\Big|_{z=1} =& \frac{z^2 K_{\bar{s}}}{K_{\bar{s}}+K_s}\partial_z\Bigg[\Big(\left(A_s\right)^2\ {}_1F_1\left(\cdot,\cdot,\Delta A_s(z-1)\right) + \frac{2\Delta A_s K_s A_s}{1+K_s+K_{\bar{s}}}\ {}_1F_1\left(+,+,\Delta A_s(z-1)\right) \\ &+ \frac{(\Delta A_s)^2 K_s\left(K_s+1\right)}{(1+K_s+K_{\bar{s}})(2+K_s+K_{\bar{s}})} \cdot\ {}_1F_1\left(++,++,\Delta A_s(z-1)\right)\Big) e^{A_s(z-1)}\Bigg]\Bigg|_{z=1}. \end{aligned} \tag{S25} $$

Differentiating and using property (S23), we obtain

$$ \begin{aligned} \left(z^2\partial_z^3 G_s(z)\right)\Big|_{z=1} =& \frac{z^2 K_{\bar{s}}}{K_{\bar{s}}+K_s} e^{A_s(z-1)}\Big[\left(A_s\right)^3\ {}_1F_1\left(\cdot,\cdot,\Delta A_s(z-1)\right) \\ &+ \frac{3\Delta A_s K_s (A_s)^2}{1+K_s+K_{\bar{s}}}\ {}_1F_1\left(+,+,\Delta A_s(z-1)\right) \\ &+ \frac{3(\Delta A_s)^2 K_s\left(K_s+1\right)K_s}{(1+K_s+K_{\bar{s}})(2+K_s+K_{\bar{s}})}\ {}_1F_1\left(++,++,\Delta A_s(z-1)\right) \\ &+ \frac{(\Delta A_s)^3 K_s\left(K_s+1\right)\left(K_s+2\right)}{(1+K_s+K_{\bar{s}})(2+K_s+K_{\bar{s}})(3+K_s+K_{\bar{s}})}\ {}_1F_1\left(+++,+++,\Delta A_s(z-1)\right)\Big]\Big|_{z=1}. \end{aligned} \tag{S26} $$

Evaluating at $z=1$, we obtain

$$ \left(z^2\partial_z^3 G_s(z)\right)\Big|_{z=1} = \frac{K_{\bar{s}}}{K_{\bar{s}}+K_s}\left[\left(A_s\right)^3 + \frac{\Delta A_s K_s}{1+K_s+K_{\bar{s}}}\left(3\left(A_s\right)^2 + \frac{\Delta A_s(K_s+1)}{2+K_s+K_{\bar{s}}}\left(3A_s + \frac{\Delta A_s(K_s+2)}{3+K_s+K_{\bar{s}}}\right)\right)\right]. \tag{S27} $$

Finally, summing on the possible states of s we arrive at Eq. (S22). □

SMALL-NOISE APPROXIMATION TO RAMP SENSING

Input noise

In the Model section of the main text, we built a model for a single receptor/ion channel that encodes information from an cell-external environment in some cell-internal degrees of freedom. Similarly to [23], we assume that the receptor/ion channel activity ($r(t)$) is a two state system: *on* with $r=1$ when the receptor is bound or the channel open, and *off* with $r=0$ when the receptor is not bound or the channel is closed. The external concentration ($c(t)$) is assumed to affect the unbound/closed time interval $\langle\tau_u\rangle = [k_+ c(t)]^{-1}$ but not the bound/open time internal $\langle\tau_b\rangle = k_-^{-1}$, where k_+ and k_- are both constants. Both interval durations are assumed to be independent, exponentially distributed random variables. The independence of binding and unbinding (or equivalently of opening and closing)

means that the probability of a molecule binding the receptor a second time is negligible. We therefore assume the system to be in the fast diffusion regime.

The signaling rate, called u, implements two different mechanisms of encoding, either continuous (CM) or bursty (BM) modulation. CM and BM ultimately correspond to amplitude (AM) and frequency (FM) modulation, respectively, when generalized to multiple receptors/ion channels as explained in the Results section of the main text. In CM the proteins are produced with a constant rate α during the binding time. On the other hand, for BM a burst of ζ proteins is realized at the time of binding, so

$$u(t) = \begin{cases} \alpha\ r(t) & \text{for CM} \\ \zeta \sum \delta(t - t_i^+) & \text{for BM,} \end{cases} \tag{S28}$$

where $\alpha k_-^{-1} = \zeta$ and t_i^+ the binding times. By taking the average of the rate $u(t)$ over a time $\tilde{t}$ much longer than both the average bound time, $\langle \tau_b \rangle = k_-^{-1}$, and the average unbound time, $\langle \tau_u \rangle = [k_+ c(t)]^{-1}$, but shorter than the time during which the external concentration changes, we obtain

$$\langle u(t) \rangle = \zeta \frac{k_+ c(t)}{1 + k_+ c(t)/k_-}, \tag{S29}$$

$$\langle \delta u(t) \rangle = 0,$$

$$\langle \delta u(t) \delta u(t') \rangle = g \zeta^2 \frac{k_+ c(t)}{(1 + k_+ c(t)/k_-)^3} \delta(\ t - t'), \tag{S30}$$

$$g = \begin{cases} 2 & \text{for CM} \\ 1 + [k_+ c(t)/k_-]^2 & \text{for BM,} \end{cases} \tag{S31}$$

which become Eqs. (3), (4) and (5) in the main text by setting $\zeta = 1$. (See Supplementary Information in [23] for further details). Importantly, $g_{\rm BM} < g_{\rm CM}$ since $k_+ c(t) < k_-$, *i.e.* $\langle \tau_b \rangle < \langle \tau_u \rangle$.

By considering an external concentration given by Eq. (6) in the main text,

$$c(t) = \begin{cases} c_0 + c_1 t, & t \geq 0 \\ c_0, & t < 0, \end{cases}$$

with $c_1 t \ll c_0$, Eq. (S29) becomes

$$u(t) = \zeta \begin{cases} u_0 + u_1 t + \delta u(t), & t \geq 0 \\ u_0 + \delta u(t), & t < 0, \end{cases} \tag{S32}$$

where we assume $\delta u(t) \ll u_0 + u_1 t$ and

$$u_0 = \frac{k_+ c_0}{(1 + k_+ c_0/k_-)}, \tag{S33}$$

$$u_1 = \frac{k_+ c_1}{(1 + k_+ c_0/k_-)^2}, \tag{S34}$$

$$\langle \delta u(t) \delta u(t') \rangle_{t,t' \geq 0} = \begin{cases} \frac{\delta(t-t')}{(1+k_+ c_0/k_-)^2} \left[g_{\rm CM} u_0 + g_{\rm CM} \left(1 - 2 k_+ c_0/k_-\right) u_1 t \right] & \text{for CM} \\ \frac{\delta(t-t')}{(1+k_+ c_0/k_-)^2} \left[g^*_{\rm BM} u_0 + \left(1 - 2k_+ c_0/k_- + 3 k_+^2 c_0^2/k_-^2\right) u_1 t \right] & \text{for BM,} \end{cases} \tag{S35}$$

$$\langle \delta u(t) \delta u(t') \rangle_{t,t' < 0} = \frac{g u_0}{(1 + k_+ c_0/k_-)^2} \delta(t - t'). \tag{S36}$$

Here $g_{\rm CM} = 2$ and $g^*_{\rm BM} = 1 + (k_+ c_0/k_-)^2$ (cf. Eq. (8) in the main text). Again for $\zeta = 1$, this becomes Eq. (7) in the main text. From Eqs. (S32)-(S36), the constant ($t < 0$) and ramp ($t \geq 0$) regimes for the external species c are encoded in the rate u in the corresponding regimes since the condition $c_1 t \ll c_0$ ensures $u_1 t \ll u_0$. However, to satisfy condition $\delta u \ll \langle u \rangle$ for both CM and BM, a new condition is needed:

$$\frac{g_{\rm CM/BM}}{k_+ c_0 (1 + k_+ c_0/k_-)} \ll \tau_c, \tag{S37}$$

which implies

$$k_-,\ k_+ c_0 \gg \tau_c^{-1}. \tag{S38}$$

Here, we have introduced the correlation time of white noise, τ_c, corresponding to the δ-function used in Eqs.(S35) and (S36). Note that condition in (S38) restricts our study to the fast switching regime. Finally, the signaling rate u in the constant regime has one small term $\delta u/u_0$ of order δ which is defined by Eqs. (S32) and (S36). Instead, small-ramp regime u contains two small terms: the small-ramp term $u_1 t/u_0$ of order ϵ and the small noise term $\delta u/u_0$ which now has a correction to order δ coming from the small ramp (order ϵ). With these definitions, from Eqs. (S32)-(S35) the rate u in the small ramp regime has two small corrections to the constant rate u_0

$$u(t) = \zeta u_0 \left(1 + \underbrace{\frac{u_1 t}{u_0}}_{o(\epsilon)} + \underbrace{\frac{\delta u}{u_0}}_{o\left(\delta(1+\sqrt{\epsilon})\right)} \right), \tag{S39}$$

In order to linearize around linear solutions, we further assume that the small-noise amplitude is smaller than the small ramp. As a result, $o(\delta) \sim o(\epsilon^x)$ with $x > 1$, which means that

$$g(1 + k_+ c_0/k_-)k_+ c_0 < \tau_c \left(k_+ c_1 t\right)^2 . \tag{S40}$$

Note that for simplicity, both in the following sections and in the main text, we set $\zeta = 1$.

Output noise in incoherent feedforward loop

Average solutions for ramp sensing

Eqs. (9) and (10) in the main text for the incoherent feedforward loop for $f(u) = e^{bu}$ and $g(y) = e^{-bk_y y}$ become

$$\frac{dx}{dt} = k_x \left[e^{b(u-k_y y)} - x \right], \tag{S41}$$

$$\frac{dy}{dt} = u - k_y y. \tag{S42}$$

Here, b is a constant introduced to maintain the exponent unitless, and k_x and k_y are rate constants for x and y. This system of equations performs exact adaptation. The steady-state solution in the constant regime ($t < 0$ in Eq. (S32)) is

$$\langle x(t) \rangle = 1, \qquad \langle y(t) \rangle = \frac{u_0}{k_y}, \tag{S43}$$

which sets the initial conditions $\langle x(0) \rangle = 1$ and $\langle y(0) \rangle = u_0/k_y$ for Eqs. (S41) and (S42) in the ramp regime ($t \geq 0$ in Eq. 7). With these initial conditions, the solutions for $t \geq 0$ can be written as

$$\langle x(t) \rangle = e^{-k_x t} k_x e^{\frac{bu_1}{k_y}} \int_0^t dt' e^{k_x t' - \frac{bu_1}{k_y} e^{-k_y t'}}, \tag{S44}$$

$$\langle y(t) \rangle = \frac{u_0}{k_y} - \frac{u_1}{k_y^2} + \frac{u_1}{k_y} t + \frac{u_1}{k_y^2} e^{-k_y t}, \tag{S45}$$

where the integral in the expression for $\langle x(t) \rangle$ cannot be solved analytically. However, by assuming that the integral starts from time $\epsilon \gg k_y^{-1}$, Eq. (S44) becomes

$$\langle x(t) \rangle \simeq e^{\frac{bu_1}{k_y}} + e^{-k_x(t-\epsilon)} \left[\langle x(\epsilon) \rangle - e^{\frac{bu_1}{k_y}} \right].$$

Finally, by considering t such that $t - \epsilon \gg k_x^{-1}$ and without exceeding the small-ramp regime (*e.g.* $k_{x,y} \gg 1$ and ϵ small), the solution becomes Eqs. (13a-b) in the main text,

$$\langle x(t) \rangle = e^{\frac{bu_1}{k_y}},$$

$$\langle y(t) \rangle = \frac{u_0}{k_y} - \frac{u_1}{k_y^2} + \frac{u_1}{k_y} t.$$

These solutions match numerical results shown in S4A Fig. Note that the time interval over which these solutions are valid extends from a time larger than the transient time to around a time that does not exceed the small-ramp regime. These criteria also set the regime of validity for our next results.

Output variances in ramp sensing

Above we gave the average solutions for the incoherent feedforward loop, both in the constant regime ($t < 0$, Eq. (S43)) and in the ramp regime ($t \geq 0$, Eqs. (S44) and (S45)). Now we want to linearize the equations around these solutions in order to obtain information about the noise. We assume that the input noise (δu) is smaller than the ramp, Eqs. (S38) and (S40), which translates into small output noise (δx and δy). In addition to these assumptions, we also assume $b \sim u_0^{-1}$ in order to ensure $b(\delta u - k_y \delta y) \ll 1$. Hence, in the ramp regime, the differential equations for δx and δy become

$$\frac{d(\delta x)}{dt} = k_x \left[b e^{\frac{u_1 b}{k_y}} (\delta u - k_y \delta y) - \delta x \right], \tag{S47}$$

$$\frac{d(\delta y)}{dt} = \delta u - k_y \delta y. \tag{S48}$$

By defining $X(t) = \begin{bmatrix} \delta x \\ \delta y \end{bmatrix}$, Eqs. (S47) and (S48) can be rewritten in a compact way for both the constant and small-ramp regimes as

$$\frac{dX(t)}{dt} + M(t)X(t) = \begin{bmatrix} A(t)\delta u(t) \\ \delta u(t) \end{bmatrix}, \tag{S49}$$

where

$$M(t) = \begin{cases} \begin{bmatrix} k_x & k_x k_y b \\ 0 & k_y \end{bmatrix}, & t < 0 \\ \begin{bmatrix} k_x & k_x k_y b \ e^{\frac{b u_1}{k_y}} \\ 0 & k_y \end{bmatrix}, & t \geq 0, \end{cases} \tag{S50}$$

and

$$A(t) = \begin{cases} k_x b, & t < 0 \\ k_x b \ e^{\frac{b u_1}{k_y}}, & t \geq 0. \end{cases} \tag{S51}$$

Note that $M(t)$ (for $t > 0$) is independent of time, which allows Eq. (S49) to be solved analytically. This is due to our choice of $f(u)$ and $g(u)$ in Eqs. (9) and (10) in the main text.

For the constant input regime, $t < 0$, the solutions for CM and BM are

$$\left\langle (\delta x(t))^2 \right\rangle = \frac{g_{\mathrm{CM/BM}} b^2 k_x^2 u_0}{2(k_x + k_y)(1 + k_+ c_0/k_-)^2}, \tag{S52}$$

$$\left\langle (\delta y(t))^2 \right\rangle = \frac{g_{\mathrm{CM/BM}} u_0}{2k_y(1 + k_+ c_0/k_-)^2}, \tag{S53}$$

where $g_{\mathrm{CM}} = 2$ and $g_{\mathrm{BM}} = g^*_{\mathrm{BM}} = 1 + (k_+ c_0/k_-)^2$. Hence, in the constant regime the output noise for BM is lower than the output noise for CM since $k_+ c_0 < k_-$.

For the small-ramp regime, $t \geq 0$, Eq. (S49) is analytically solvable for $t \gg k_y^{-1}$ by evaluating the integral from time $\epsilon \gg k_y^{-1}$ to some time t that does not exceed the small-ramp approximation (as discussed for the average solutions). With these assumptions and by using an appropriate integrating factor, the solution for $X(t)$ is

$$\int_\epsilon^t e^{Mt'} \frac{dX(t')}{dt'} dt' + \int_\epsilon^t e^{Mt'} M X(t') dt' = \int_\epsilon^t e^{Mt'} \begin{bmatrix} A\delta u(t') \\ \delta u(t') \end{bmatrix} dt',$$

$$X(t) = e^{-Mt} \int_\epsilon^t e^{Mt'} \begin{bmatrix} A\delta u(t') \\ \delta u(t') \end{bmatrix} dt' + e^{-M(t-\epsilon)} X(\epsilon).$$

However, for $t - \epsilon \gg k_{x,y}^{-1}$ (within the limit for t and ϵ as discussed for Eqs. (13a,b) the solution is

$$X(t) = e^{-Mt} \int_\epsilon^t e^{Mt'} \begin{bmatrix} A\delta u(t') \\ \delta u(t') \end{bmatrix} dt'. \tag{S54}$$

By using matrix diagonalization, expressing the noise in u by a delta function in time (Eq. S35), and integrating Eq. (S54) for $X(t)^2$, we find analytical solutions for the variances. The results for CM are

$$\left\langle (\delta x(t))^2 \right\rangle_{\mathrm{CM}} = \frac{b^2 k_x^2 e^{\frac{2bu_1}{k_y}}}{2(k_x+k_y)(1+k_+c_0/k_-)^2} \left[g_{\mathrm{CM}} u_0 - g_{\mathrm{CM}} \frac{(1-2k_+c_0/k_-)}{k_x+k_y} u_1 + g_{\mathrm{CM}}(1-2k_+c_0/k_-)u_1 t \right], \tag{S55}$$

$$\left\langle (\delta y(t))^2 \right\rangle_{\mathrm{CM}} = \frac{1}{2k_y(1+k_+c_0/k_-)^2} \left[g_{\mathrm{CM}} u_0 - g_{\mathrm{CM}} \frac{(1-2k_+c_0/k_-)}{ky} u_1 + g_{\mathrm{CM}}(1-2k_+c_0/k_-)u_1 t \right], \tag{S56}$$

where $g_{\mathrm{CM}} = 2$. For BM, the g_{BM} parameter (see Eq. (5)) affects the integration, and the results are

$$\left\langle (\delta x(t))^2 \right\rangle_{\mathrm{BM}} = \frac{b^2 k_x^2 e^{\frac{2bu_1}{k_y}}}{2(k_x+k_y)(1+k_+c_0/k_-)^2} \left[g^*_{\mathrm{BM}} u_0 - \frac{1-2k_+c_0/k_- + 3k_+^2c_0^2/k_-^2}{k_x+k_y} u_1 + \left(1-2k_+c_0/k_- + 3k_+^2c_0^2/k_-^2\right) u_1 t \right], \tag{S57}$$

$$\left\langle (\delta y(t))^2 \right\rangle_{\mathrm{BM}} = \frac{1}{2k_y(1+k_+c_0/k_-)^2} \left[g^*_{\mathrm{BM}} u_0 - \frac{1-2k_+c_0/k_- + 3k_+^2c_0^2/k_-^2}{2ky} u_1 + \left(1-2k_+c_0/k_- + 3k_+^2c_0^2/k_-^2\right) u_1 t \right], \tag{S58}$$

where $g^*_{\mathrm{BM}} = 1 + (k_+c_0/k_-)^2$. Note that for $c_1 = u_1 = 0$, the solutions coincide with the solutions for the constant regime. Furthermore, by comparing the time-dependent terms, CM is noisier than BM when $k_+c_0 < \frac{1}{3}k_-$. In our model this is due to the input noise (cf. Eq. S35). These two regimes in which BM is less noisy and hence more accurate than CM depend on the ratio of binding and unbinding rates as shown in S4B,C Fig. Clearly the analytic solutions match numerical simulations with noise. All these calculations were done using Wolfram Mathematica 8, while all the simulations of the stochastic differential equations (Eqs. S41 and S42) were done using the Euler method in MATLAB.

Output noise for integral feedback loop

A similar approach can be applied to the integral feedback loop given by Eqs. (11) and (12) in the main text, shown here for clarity with $f(y) = e^{-by}$:

$$\frac{dx}{dt} = ue^{-by} - k_x x, \tag{S59}$$

$$\frac{dy}{dt} = k_y (x-1). \tag{S60}$$

Assuming that u is given by Eq. (S32), this system does not have analytic solutions in the ramp regime. However, in the small-ramp regime it is possible to linearize around the solutions of the constant regime. Hence, $\langle x(t)\rangle = x_0 + \epsilon_x(t)$ and $\langle y(t)\rangle = y_0 + \epsilon_y(t)$, where $x_0 = 1$ and $y_0 = \frac{\ln(u_0/k_x)}{b}$ are the solutions for the constant regime with $\langle u\rangle = u_0$. Note that the condition $k_x < u_0$ is required. By linearization, Eqs. (11) and (12) become

$$\frac{d\epsilon_x(t)}{dt} = k_x \frac{u_1 t}{u_0} - k_x\epsilon_x(t) - k_x b \epsilon_y(t) - bk_x \frac{u_1}{u_0}\epsilon_y(t)t, \tag{S61}$$

$$\frac{d\epsilon_y(t)}{dt} = k_y \epsilon_x(t). \tag{S62}$$

Combining both equations and neglecting the second-order term $u_1 t/u_0 \epsilon_y(t)$, it possible to find a second-order differential equation for $\epsilon_x(t)$, given by

$$\frac{d^2\epsilon_x(t)}{dt^2} + k_x \frac{d\epsilon_x(t)}{dt} + bk_xk_y\epsilon_x(t) = \frac{k_x u_1}{u_0}. \tag{S63}$$

The solution is

$$\epsilon_x(t) = \frac{u_1}{bk_yu_0} + C_1 \exp\left[\left(-\frac{k_x}{2} - \sqrt{\frac{k_x^2}{4} - bk_xk_y}\right)t\right] + C_2 \exp\left[\left(-\frac{k_x}{2} + \sqrt{\frac{k_x^2}{4} - bk_xk_y}\right)t\right], \tag{S64}$$

with $\epsilon_x(t) \to \frac{u_1}{bk_y u_0}$ after a transient time defined by the exponential terms for any $k_x^2/4 - bk_x k_y$. Furthermore, there are two integration constants C_1 and C_2. From Eq. (S62) we obtain $\epsilon_y = \frac{u_1}{bu_0}t - \frac{u_1}{b^2 k_y u_0}$. Finally, the solutions of linearized Eqs. (11) and (12) in the small-ramp regime after the transient time are [23]

$$\langle x(t) \rangle = 1 + \frac{u_1}{bk_y u_0}, \tag{S65}$$

$$\langle y(t) \rangle = y_0 - \frac{u_1}{b^2 k_y u_0} + \frac{u_1}{bu_0}t. \tag{S66}$$

Within the small-noise approximation (Eq. S38), we want to find expressions for the variances. In the constant regime, $u(t) = u_0 + \delta u(t)$ ($t < 0$ in Eq. 5) implies $x(t) = 1 + \delta x(t)$ and $y(t) = y_0 + \delta y(t)$. Therefore, the equations for the noise terms become

$$\frac{d(\delta x)}{dt} = k_x \left[\frac{\delta u}{u_0} - bu_0 \delta y - \delta x \right], \tag{S67}$$

$$\frac{d(\delta y)}{dt} = k_x \delta x. \tag{S68}$$

Proceeding similarly to the incoherent feedforward loop, in the constant regime the solution for the variances are [23]

$$\left\langle (\delta x)^2 \right\rangle = \frac{g_{\mathrm{CM/BM}} k_x}{2\left(1 + k_+ c_0/k_-\right)^2 u_0}, \tag{S69}$$

$$\left\langle (\delta y)^2 \right\rangle = \frac{g_{\mathrm{CM/BM}} k_y}{2b\left(1 + k_+ c_0/k_-\right)^2 u_0}, \tag{S70}$$

with $g_{\mathrm{CM}} = 2$ and $g_{\mathrm{BM}} = g^*_{\mathrm{BM}} = 1 + (k_+ c_0/k_-)^2$. Hence, in the constant regime, the output noise for BM is lower than the output noise for CM (since $k_+ c_0 < k_-$).

To study the system in the small-ramp regime ($t > 0$ in Eq. 5), we assume that the input noise (δu) is smaller than the ramp (Eqs. S38 and S40), which translates into small output noise (δx and δy), and linearize around solutions (S65) and (S66). As a result, Eq. (11) becomes

$$\frac{d\left(\delta x\right)}{dt} = k_x \left[\left(1 + \frac{u_1 t}{u_0} + \frac{\delta u}{u_0}\right)\left(1 - b\left(\epsilon_y + \delta y\right) + \frac{b^2}{2}\left(\epsilon_y + \delta y\right)^2\right) - 1 - \epsilon_x - \delta x \right],$$

which, by using Eq. (S61), becomes

$$\frac{d\left(\delta x\right)}{dt} = k_x b \left[\frac{\delta u}{u_0} - \delta y - \frac{u_1}{u_0}\epsilon_y t - \frac{u_1 t}{u_0}\delta y - \frac{\epsilon_y}{u_0}\delta u + \frac{b\epsilon_y^2}{2} + b\epsilon_y \delta y - \frac{\delta x}{b} + o(\epsilon^3, \delta^2, \epsilon^2\delta) \right], \tag{S71}$$

where we neglect third-order terms in the small ramp ($o(\epsilon^3)$), second-order terms in the small noise (δ^2) and mixed-order terms ($o(\epsilon^2\delta)$) due to the assumption that the noise is smaller than the ramp (cf. discussion that leads to Eq. (S40)).

Defining $X(t) = \begin{bmatrix} \delta x \\ \delta y \end{bmatrix}$, Eqs. (S71) and (S60) become

$$\frac{dX(t)}{dt} + M(t)X(t) = \begin{bmatrix} A(t)\delta u(t) + B(t) \\ 0 \end{bmatrix}, \tag{S72}$$

where from Eqs. (S71) and (S60), using definitions of ϵ_x and ϵ_y, $M(t) = \begin{bmatrix} k_x & k_x b\left(1 + \frac{bu_1}{k_y u_0}\right) \\ -k_y & 0 \end{bmatrix}$, $A(t) = \frac{k_x}{u_0}\left(1 + \frac{u_1}{k_y b u_0} - \frac{u_1}{u_0}t\right)$ and $B(t) = \frac{k_x u_1^2}{u_0^2}\left(\frac{t}{k_y} - t^2\right)$. Using an integrating factor and integrating between ϵ and t gives

$$X(t) = e^{-M(t-\epsilon)}X(\epsilon) + e^{-Mt}\int_\epsilon^t dz\ e^{Mz}A(z)\begin{bmatrix} \delta u \\ 0 \end{bmatrix} + e^{-Mt}\int_\epsilon^t dz\ e^{Mz}B(z)\begin{bmatrix} 1 \\ 0 \end{bmatrix}, \tag{S73}$$

where the term $e^{-M(t-\epsilon)}X(\epsilon)$ is negligible for $(t-\epsilon) \gg k_{x,y}^{-1}$. To calculate the variances we square Eq. (S73). Using Eqs. (S5)-(S9), the results for $\left\langle (\delta x(t))^2 \right\rangle$ to first-order in the small-ramp parameters are

$$\left\langle (\delta x(t))^2 \right\rangle_{CM} = \frac{kx}{2\left(1+k_+c_0/k_-\right)^2 u_0}\left[g_{\mathrm{CM}} - g_{\mathrm{CM}}\left(1+2k_+c_0/k_-\right)\frac{u_1 t}{u_0} + g_{\mathrm{CM}}\frac{2k_x + bk_y\left(1+2k_+c_0/k_-\right)u_1}{bk_yk_xu_0}\right], \quad \text{(S74)}$$

$$\left\langle (\delta x(t))^2 \right\rangle_{BM} = \frac{kx}{2\left(1+k_+c_0/k_-\right)^2 u_0}\left[g^*_{\mathrm{BM}} - \left(1+2k_+c_0/k_- - k_+^2c_0^2/k_-^{-2}\right)\frac{u_1 t}{u_0} + \frac{\left(bk_y\left(1+2k_+c_0/k_- - k_+^2c_0^2/k_-^{-2}\right) + 2k_xg^*_{\mathrm{BM}}\right)u_1}{bk_xk_yu_0}\right]. \quad \text{(S75)}$$

Similarly the results for $\left\langle (\delta y)^2 \right\rangle$ are

$$\left\langle (\delta y(t))^2 \right\rangle_{CM} = \frac{k_y}{2b\left(1+\langle\tau_b\rangle k_+c_0\right)^2 u_0}\left[g_{\mathrm{CM}} - g_{\mathrm{CM}}(1+2k_+c_0/k_-)\frac{u_1 t}{u_0} + g_{\mathrm{CM}}\frac{(kx(3+2k_+c_0/k_-) + 2k_yb(1+2k_+c_0/k_-))\,u1}{2bk_xk_yu_0}\right], \quad \text{(S76)}$$

$$\left\langle (\delta y(t))^2 \right\rangle_{BM} = \frac{k_y}{2b\left(1+\langle\tau_b\rangle k_+c_0\right)^2 u_0}\left[g^*_{\mathrm{BM}} - \left(1+2\langle\tau_b\rangle k_+c_0 - \langle\tau_b\rangle^2 k_+^2c_0^2\right)\frac{u_1 t}{u_0} + \frac{\left[2bk_y\left(1+2\langle\tau_b\rangle k_+c_0 - \langle\tau_b\rangle^2k_+^2c_0^2\right) + k_x\left(3+2\langle\tau_b\rangle k_+c_0 + \langle\tau_b\rangle^2k_+^2c_0^2\right)\right]u_1}{bk_yk_xu_0}\right], \quad \text{(S77)}$$

where $g^*_{\mathrm{BM}} = 1 + (k_+c_0/k_-)^2$. Note that for $c_1 = u_1 = 0$, the solutions coincide with the solutions for the constant regime. Although it is clear that BM is less noisy than CM for $k_+c_0 < k_-$, by comparing the time-dependent terms we find that, in fact, BM is always less noisy than CM. The analytical solutions are plotted in S5 Fig. and match the numerical simulations with noise. Again, all these calculations were done using Wolfram Mathematica 8, while all the simulations of the stochastic differential equations (Eqs. S59 and S60) were done using the Euler method in MATLAB.

FURTHER INVESTIGATIONS INTO THE ACCURACY

In this section we provide further explanations for the accuracy of concentration sensing by a single receptor without comparing with the maxmimum-likelihood estimation [44]. In Fig. 5 we showed results from the master equation for the two regimes $k_+c_0 < k_-$ and $k_+c_0 > k_-$ for slow and fast switching of the receptor. Despite its burstiness, the BM receptor turned out more accurate than the CM receptor in the $k_+c_0 < k_-$ regime for fast switching.

Additional results from the master-equation model. To understand this result better we also implemented an intermediate-modulation (IM) receptor, which has features of both the CM and BM receptors. Like the CM receptor, the IM receptor signals while in the bound (*on*) state, but instead of a constant rate α of production it produces protein with a rate α' so that in each bound interval the same number of molecules are produced irrespective of the interval length, i.e. $\alpha'\tau_b = \zeta$, with ζ the constant burst size of BM. For this to work, the IM receptor would have to know at the time of binding when it will unbind again, in order to choose the correct rate of production. Since the rate of unbinding is a random variable this is generally not possible. Nevertheless, the IM receptor may help to further elucidate our observed trends in accuracy. In practice, we implemented this IM receptor by first simulating a time trace of bound and unbound time intervals with a Gillespie algorithm, allowing us to determine the rate of production as a function of time. Afterwards, the actual protein production and degradation were simulated.

In analogy to Fig. 5 the results for the IM receptor are shown in S1 Fig. (green lines), which also shows the results for the CM and BM receptors for comparison in blue and red, respectively. As expected, for slow switching the IM receptor has intermediate accuracy between CM and BM. CM is most accurate as continuous production during the bound intervals is balanced by degradation so the output protein level does not fluctuate excessively. BM is least accurate due to the increased burst size for slow switching. Since signaling by the IM receptor is only burst-like for the short bound intervals but not for the long bound intervals, it is somewhat more accurate than BM. Due to the

non-constant rate of production, IM also fluctuates more than CM. This intermediate accuracy is clearly demonstrated by the time traces in the left panels of S2 Fig.

In the $k_+c_0 < k_-$ regime for fast switching, the inset of S1C Fig. shows that BM is now most accurate and that IM has again intermediate accuracy. While BM steadily produces the same amount of protein at the times of binding, IM produces this amount only during short bound intervals as its rate of production is then high, while during long bound intervals its slow production is buffered by degradation, so its protein level fluctuates more strongly. CM is even worse than IM since, due to its constant rate of production during bound time intervals, it hardly produces any protein during short bound intervals, which leads to drastic drops in protein level, while it produces a lot during long bound intervals due to its constant rate of production.

In contrast, in the $k_+c_0 > k_-$ regime for fast switching, CM is generally most accurate due to its approximately constant rate of production throughout time, i.e. the receptor is almost always bound and active. IM is less accurate than CM because its rate of protein production is variable due to the variable length in bound intervals, despite the fact that the receptor is mostly bound. Interestingly, IM is even less accurate than BM under these conditions. Inspecting the examples of time trace in the bottom right panel of S2 Fig., the burst sizes of IM can exceed the burst sizes of BM for unusually short bound intervals since production is very high and stochastic, and only on average the same amount of protein is produced during bound intervals than during a burst in BM. During long bound intervals the rate of production is very low. Hence, compared to BM, degradation prevents a net increase in protein level during a bound interval, leading to further variability. A special case is when the burst size ζ is 1. As shown in the inset of Fig. 5D, BM can be more accurate than CM. This is because the burst size of BM is minimal and in the master equation the production with minimal rate α in CM is highly stochastic.

As we now discuss, to provide further intuition for the differences in accuracy between the $k_+c_0 < k_-$ and $k_+c_0 > k_-$ regimes, we also simulated the variance of the signaling output (and hence the accuracy-determining factor g) directly (see Eq. 5).

Signaling output from ODE model without protein production and degradation. Factor g in Eq. 5 (and Eq. S31) determines the variance of the signaling rate $u(t)$ without invoking any downstream protein production and degradation. For a given time interval Δt, we can hence simulate $u(t)$ directly. We assess the accuracy of CM, IM, and BM by plotting the histograms of the integrated signaling rate $u_I(\Delta t) := \int_0^{\Delta t} u(t)\mathrm{d}t$ and by determing their variances (cf. derivation of g in [2]). As slow protein production and degradation strongly affect the accuracy of the final protein output for slow switching, this approach mainly helps understand the interesting fast switching case.

We initially assume signaling during bound intervals is deterministic, leading to a linear increase of u with slope α (α') during a bound time interval for CM (IM) and a step increase by ζ for BM. At each unbound time interval, IM and BM have the same level of signaling output as IM produces the same number of proteins deterministically during each bound interval (ζ). In contrast, the signaling output from CM is generally different since the rate of signaling is always the same for each bound interval but their durations vary. Resulting time traces and variances are shown in S3A and B Figs. left panels, respectively. Specifically, S3A Fig., left panels shows clearly that for $k_+c_0 < k_-$ BM and IM are most accurate with $u_I(t)$ increasing almost linearly in time. Since signaling is deterministic, BM and IM are essentially identical, and their variance may only differ due to small differences in signaling during the final bound interval (S3A Fig., bottom left panel). This last bound time interval may be interrupted in IM, but for long Δt this difference is negligible. In contrast, S3B Fig., left panels show clearly that for $k_+c_0 > k_-$ CM is most accurate, as $u_I(t)$ is now almost linear in time.

Signaling output from master-equation model without protein production and degradation. Allowing signaling to be stochastic does not change the results for the accuracy significantly. S3A Fig. right panels show that for $k_+c_0 < k_-$ BM is now most accurate and that IM has intermediate accuracy (between BM and CM) due to its variability in signaling in line with S1C Fig. Additionally, S3B Fig., right panels show that CM is still most accurate but also that IM is worse than BM in line with S1D Fig.

Taken together, these additional simulation results confirm our findings of the main text that BM is most accurate for $k_+c_0 < k_-$ and CM is generally most accurate for $k_+c_0 > k_-$.

AM IS MORE ACCURATE THAN FM FOR MULTIPLE RECEPTORS/ION CHANNELS

Here, we provide a more detailed discussion of the accuracy of encoding by multiple receptors, i.e. using AM and FM. To determine whether AM or FM is more accurate in encoding and decoding, we generalize to multiple receptors (or ion channels) (S6 Fig.). We assume that AM is obtained by unsynchronized CM receptors (S6A Fig.), while FM is obtained by synchronized receptors that individually operate with BM (S6D Fig.). Other types of synchronization are also possible with synchronized CM receptors shown in S6B Fig. and unsynchronized BM receptors shown in

S6C Fig. However, these receptors exhibit imperfect FM: resulting bursts have either variable duration (S6B Fig.) or variable amplitude (S6C Fig.) in contrast to the data (Fig. 1) [10, 15].

To estimate the accuracy, we first consider perfect synchronization and unsynchronization in either modulation scheme. For N unsynchronized (us) receptors, we can express the resulting average and variance of the encoded input by the single-receptor quantities, i.e. $\langle u(t)\rangle_N^{us} = N\langle u(t)\rangle_1$ and $\langle \delta u(t)\delta u(t')\rangle_N^{us} = N\langle \delta u(t)\delta u(t')\rangle_1$. As a result, the relative variance (variance divided by the average-squared) scales with N^{-1}. In contrast, for N synchronized (s) receptors, the average and variance of the encoded input can be written as $\langle u(t)\rangle_N^{s} = N\langle u(t)\rangle_1$ and $\langle \delta u(t)\delta u(t')\rangle_N^{s} = N^2\langle \delta u(t)\delta u(t')\rangle_1$, respectively. Hence, the relative variance is now independent of N, so unsynchronized receptors have an N times smaller noise than synchronized receptors. Since N unsynchronized CM receptors lead to AM, we obtain for its relative variance

$$\frac{\langle \delta u(t)\delta u(t')\rangle_N^{AM}}{\left(\langle u(t)\rangle_N^{AM}\right)^2} = \frac{\langle \delta u(t)\delta u(t')\rangle_1^{CM}}{N\left(\langle u(t)\rangle_1^{CM}\right)^2}. \tag{S78}$$

Conversely since N synchronized BM receptors lead to FM, the relative variance of FM is

$$\frac{\langle \delta u(t)\delta u(t')\rangle_N^{FM}}{\left(\langle u(t)\rangle_N^{FM}\right)^2} = \frac{\langle \delta u(t)\delta u(t')\rangle_1^{BM}}{\left(\langle u(t)\rangle_1^{BM}\right)^2}. \tag{S79}$$

For slow dynamics, or fast dynamics with $k_+c > k_-$, CM is more accurate than BM. Hence, for N receptors, AM is even more accurate than FM. In contrast, for fast dynamics with $k_+c < k_-$, BM is up to twice as accurate as CM (Eq. (5)), and AM is N times more accurate than CM. Consequently, AM becomes more accurate for encoding than FM for more than two receptors (S6E Fig.). An exception are two receptors, for which AM and FM can be equally accurate (S6E Fig., inset). Since we generally show that larger signaling noise leads to larger output noise, the same rule emerges for decoding.

To extend our results to intermediate levels of synchronization for $N > 2$ receptors we consider a fraction ρ of synchronized receptors while the remaining fraction $(1-\rho)$ are unsynchronized, with signaling either by CM or BM (S6E Fig.). When comparing CM and BM receptors for the same levels of synchronization ρ, BM receptors can remain more accurate than CM receptors (S6E Fig.). However, intermediate levels of synchronization do not strictly represent AM and FM. As shown in S6B,C Figs. synchronized CM receptors lead to pulses of variable duration, while unsynchronized BM receptors lead to highly frequent pulses with potentially variable amplitude.

Taken together, since single cells have thousands of receptors and ion channels, AM is the most accurate modulation scheme.
