# Supplementary figures and images for: Accurate Encoding and Decoding by Single Cells: Amplitude Versus Frequency Modulation
Source: PLoS Comput Biol. 2015 Jun 1;11(6):e1004222. doi: 10.1371/journal.pcbi.1004222 (PMC4452646; doi:10.1371/journal.pcbi.1004222)

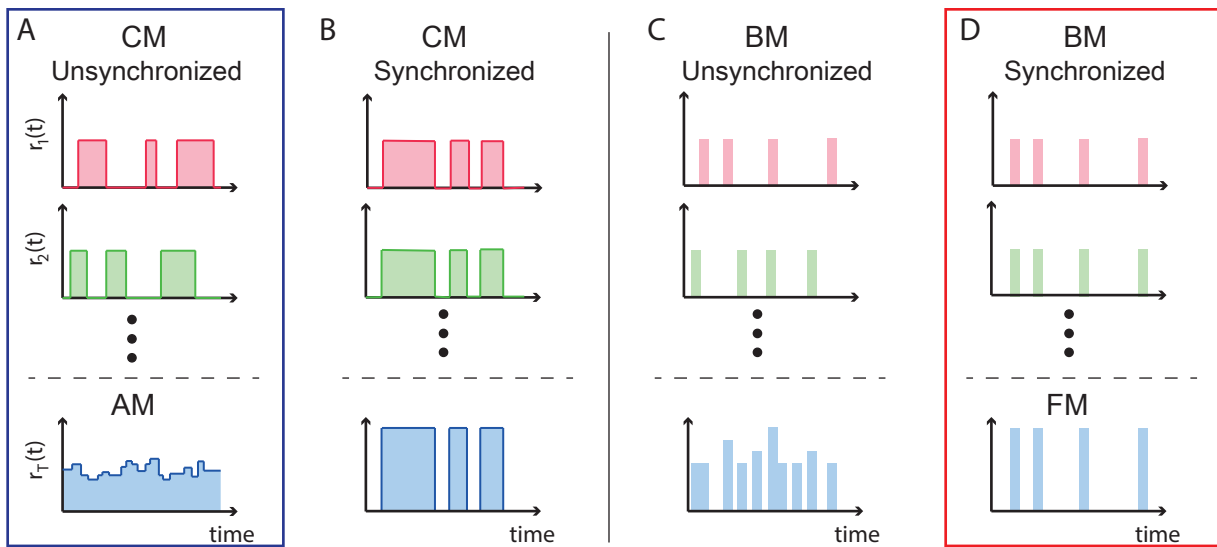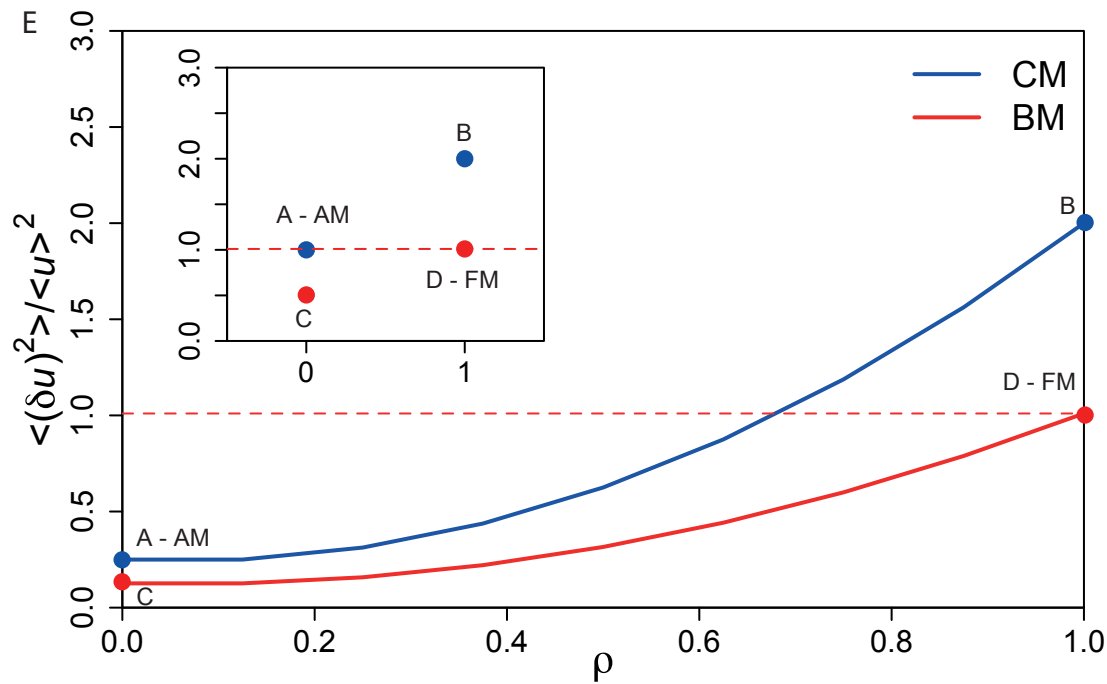

Supplement: S6 Fig — (A-D) Schematic of receptor activity in time. (A) AM emerges from N unsynchronized receptors or ion channels in CM mode. (B) N synchronized CM receptors lead to a hybrid mechanism with information encoded in the frequency of broad bursts of variable duration. (C) N unsynchronized BM receptors provide a dense series of bursts. For large N, bursts may start overlapping, leading to variable amplitudes. (D) FM emerges from N synchronized receptors in BM mode. (E) Relative variance for a system of 8 receptors with ρN synchronized and (1−ρ)N unsynchronized receptors, plotted for fast dynamics in the k + c < k − regime (CM in blue and BM in red). Letters refer to panel labels (A-D). Dotted red line indicates uncertainty from FM for comparison. (Inset) Same for a system of two receptors only. (PDF) [file pcbi.1004222.s007.pdf]
